# Construction and validation of a prediction model for complications of femoral artery access

**DOI:** 10.3389/fsurg.2025.1689625

**Published:** 2025-10-21

**Authors:** Zhifei Feng, Jiali Ding, Zhangyi Liu, Sen Tan, Chenyue Xia, Dabiao Li, Dingwei Fu, Guowu Zhang

**Affiliations:** 1 General Surgery, Yongchuan Hospital Affiliated with Chongqing Medical University, Chongqing, China; 2 Chongqing Medical University, Chongqing, China

**Keywords:** femoral artery, interventional access, postoperative complications, nomogram, risk factors

## Abstract

**Objective:**

To analyze the risk factors for the complications of access and to construct and validate a nomogram prediction model for their occurrence.

**Methods:**

Patients undergoing endovascular intervention via femoral artery access between January 2020 and April 2025 were enrolled in the study. Related clinical data were retrospectively collected and analyzed. Patients were divided into complication (*n* = 19) and non-complication (*n* = 488) groups based on the occurrence of postoperative complications associated with femoral artery puncture site. The general cohort characteristics were compared between the two groups, and the risk factors for the postoperative complications were identified based on univariate and multivariate logistic regression analyses. A nomogram prediction model was constructed and its performance was evaluated using the area under the receiver operating characteristic (ROC) curve, the Hosmer-Lemeshow test, calibration curve, and decision curve analyses.

**Results:**

Four potential predictors were identified based on the multivariate logistic regression analysis results: vascular calcification [odds ratio (OR) = 7.952, 95% confidence interval (CI): 1.653–38.254], history of diabetes (OR = 18.793, 95% CI: 3.670–96.225), platelet count (OR = 0.980, 95% CI: 0.967–0.994), and positional relationship between the puncture point and femoral head (OR = 6.125, 95% CI: 1.048–35.800). The nomogram model incorporating these factors demonstrated strong performance, with an area under the ROC curve of 0.924 (95% confidence interval: 0.839–1.000), sensitivity of 81.80%, specificity of 95.20%, and overall accuracy of 94.70%.The Hosmer-Lemeshow test yielded *χ*^2^ = 12.535 and *P* = 0.8184, indicating a good model fit. Calibration curves showed strong agreement between the nomogram predictions and observed outcomes. Both the ROC and decision curve analysis confirmed the nomogram's robust predictive performance.

**Conclusions:**

Platelet count, history of diabetes, vascular calcification, and positional relationship between the puncture point and the femoral head are independent risk factors for the complications of femoral artery access. The nomogram model established based on these indicators demonstrated a high accuracy in predicting the risk of complications.

## 1 Introduction

Over 7 million percutaneous vascular interventions are performed worldwide annually. The common femoral artery is the most commonly used target vessel for puncture due to its large diameter, superficial location, and anatomical position anterior to the femoral head. However, clinical data show that approximately 5%–10% of patients experience access site complications, such as puncture site bleeding, hematoma, pseudoaneurysm, and even retroperitoneal hematoma (1, 2). These complications severely impact patients' quality of life and increase healthcare costs. With the rising adoption of technologies, such as transcatheter aortic valve replacement and thoracic endovascular aortic repair, the growing demand for larger-diameter devices for transfemoral access has substantially heightened the potential risk of vascular complications (3). Scientific strategies for the prevention and control of vascular access complications require accurate identification of relevant risk factors. Numerous clinical studies have demonstrated that the relative position of the femoral head access site, chronic hyperglycemia, and calcification at the access site are key factors influencing the incidence of postoperative complications (4–6). However, research on risk prediction models for femoral artery access complications remains limited. The present study aimed to systematically analyze the independent risk factors for femoral artery intervention and construct and validate a prediction model that can identify high-risk groups early in order to reduce the incidence of postoperative complications.

## 2 Methods

**Figure 1:**
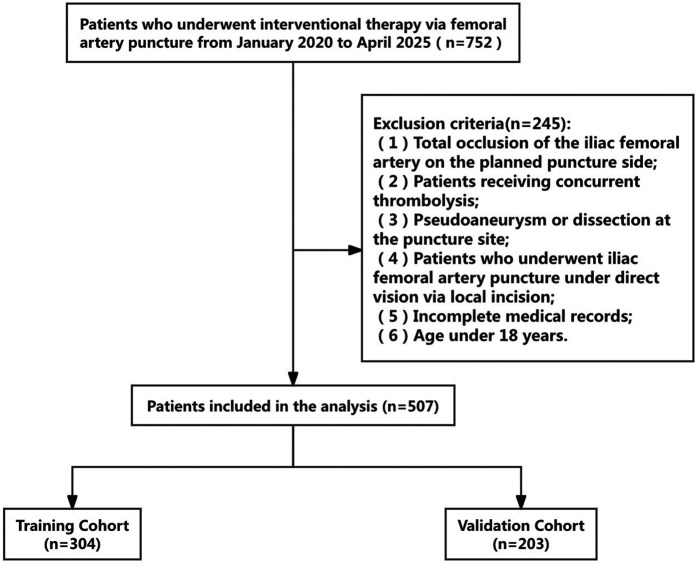
Flowchart of a screening process. A total of 507 participants were included in the analysis: 304 in the training and 203 in the validation cohorts.

1. The clinical records of 507 patients who underwent femoral artery puncture at Yongchuan Hospital Affiliated with Chongqing Medical University between January 2020 and April 2025 were retrospectively analyzed. The cohort included 398 male and 109 female patients. A flowchart of the patient selection process is shown in Figure 1. The 507 patients were randomly divided into training (*n* = 304) and validation (*n* = 203) sets at a 6:4 ratio to ensure the reproducibility of the model's training and validation processes. The demographic and clinical characteristics of all patients in the training and validation sets are represented in Table 1. This clinical study adhered to the Declaration of Helsinki and met relevant ethical requirements. The study was approved by the Medical Ethics Committee of Yongchuan Hospital Affiliated with Chongqing Medical University.
2. Inclusion criteria: (1) Age of ≥18 years; (2) femoral artery puncture access; and 3. Complete medical records; Exclusion criteria: (1) Total occlusion of the femoral artery on the planned puncture side; (2) Patients who received concurrent thrombolysis; (3) Patients with pseudoaneurysm or dissection on the puncture side; (4) Patients who received femoral artery puncture under direct visualization through a local incision; (5) Incomplete medical records; and (6) Age of <18 years.
3. The study preliminarily formulated 18 risk factors based on expert consultation and literature review. The following patient information was collected: age, sex, body mass index (BMI), Vascular calcification, thrombosis, and Vascular tortuosity, history of hypertension, history of diabetes and smoking, operation duration, Hospitalization duration, platelet count, international normalized ratio, serum creatinine level, Chronic Kidney Disease (CKD), maximum sheath size, Surgery duration, vascular closure device (VCD), and positional relationship between the puncture site and the femoral head. The patient information recorded at discharge was considered the observation endpoint. Based on the model sample size calculation formula (4), 5–10 cases were required to validate each variable.

**Table 1:** Differences in characteristics between the training and validation groups.

Variables	Total (*n* = 507)	Training group (*n* = 304)	Validation group (*n* = 203)	*t*/Z/*χ*^2^	*p*
Complication [*n* (%)]				<0.001	1
No	488 (96.25)	293 (96.38)	195 (96.06)		
Yes	19 (3.75)	11 (3.62)	8 (3.94)		
VCD [*n* (%)]				2.083	0.353
Vascular occluder	375 (73.96)	228 (75.00)	147 (72.41)		
Suture-mediated vascular Closure device	132 (26.04)	76 (25.00)	56 (27.59)		
PP—FH position relationship [*n* (%)]				0.644	0.422
Above	462 (91)	274 (90)	188 (93)		
Around	45 (9)	30 (10)	15 (7)		
Sex [n (%)]				0.036	0.85
Female	109 (21)	64 (21)	45 (22)		
Male	398 (79)	240 (79)	158 (78)		
Vascular calcification [*n* (%)]				0.262	0.609
No	426 (84)	258 (85)	168 (83)		
Yes	81 (16)	46 (15)	35 (17)		
Thrombosis [*n* (%)]				0.305	0.581
No	453 (89)	274 (90)	179 (88)		
Yes	54 (11)	30 (10)	24 (12)		
Vascular tortuosity [*n* (%)]				0.572	0.409
No	501 (99)	299 (98)	202 (100)		
Yes	6 (1)	5 (2)	1 (0)		
Hypertension [*n* (%)]				2.229	0.135
No	274 (54)	173 (57)	101 (50)		
Yes	233 (46)	131 (43)	102 (50)		
Diabetes [*n* (%)]				1.885	0.173
No	420 (83)	258 (85)	162 (80)		
Yes	87 (17)	46 (15)	41 (20)		
History of smoking [*n* (%)]				0.911	0.34
No	218 (43)	125 (41)	93 (46)		
Yes	289 (57)	179 (59)	110 (54)		
CKD [*n* (%)]				0.816	0.366
No	488 (96)	295 (97)	193 (95)		
Yes	19 (4)	9 (3)	10 (5)		
Maximum sheath size [*n* (%)]				4.897	0.724
≤8F	387 (76.33)	236 (77.63)	151 (74.38)		
>8F	120 (23.67)	68 (22.37)	52 (25.62)		
Age	64 (54.73)	63 (54.73)	65 (54.73)	−0.401	0.688
BMI	23.44 (21.14, 25.18)	23.44 (21.26, 25.4）	23.44 (20.82, 25)	−0.047	0.963
Surgery duration (min)	120 (70, 190)	120 (75, 200)	110 (70, 187.5)	−1.406	0.16
Hospitalization duration (day)	9 (6, 12.5)	9 (6, 13)	9 (6, 12)	−0.135	0.893
Platelet count	177 (124.5, 232.5)	178 (126.5, 223)	176 (124, 241)	−0.938	0.349
INR	1.05 (0.97, 1.14)	1.05 (0.98, 1.14)	1.05 (0.97, 1.16)	−0.09	0.929
Serum creatinine	68 (56, 80)	68 (55, 79.25)	68 (57, 80)	−0.127	0.9

PP—FH position relationship: the positional relationship between the puncture point and femoral head; INR, international normalized ratio; CKD, prior diagnosis of CKD was established at a tertiary hospital. Vascular calcification, the CT attenuation value of calcified foci is ≥130 Hounsfield Units. Vascular tortuosity: the “double iliac sign” was visualized on a single CT axial plane.

### 2.1 Statistical methods

Data analysis and graphs were generated using SPSS 23.0 and R packages. Continuous data were evaluated for normality using the Kolmogorov–Smirnov test. Normally distributed continuous data were expressed as ‘x ± s, and inter-group comparisons were performed using the independent sample *t*-test. Continuous data that did not follow a normal distribution were expressed as M (Q1, Q3), and the Mann–Whitney *U* test was used for inter-group comparisons. Count data were expressed as percentages (%), and the chi-square test or Fisher's exact test was used for inter-group comparisons. Univariate and multivariate logistic regression model was used to analyze the risk factors of complications. A nomogram prediction model was constructed based on the identified independent risk factors. The receiver operating characteristic (ROC) curve was utilized to determine the efficacy of the nomogram model in predicting complications. The calibration curve was employed to evaluate the model's calibration rate. The clinical decision curve was used to assess the clinical benefit of the model. A two-sided *P* < 0.05 was considered statistically significant.

### 2.2 Data collection

Two team members completed all data collection between January 2025 and April 2025. All researchers underwent standardized training and assessment to ensure the standardization of data collection and research methods and to prevent data bias or errors due to human factors. Only those who passed the assessment were allowed to participate in the study. Furthermore, data entry was performed by two persons and was double-checked to ensure its accuracy, completeness, and authenticity. Any errors or discrepancies were reported to the researchers for prompt evaluation and correction.

## 3 Results

**Figure 2:**
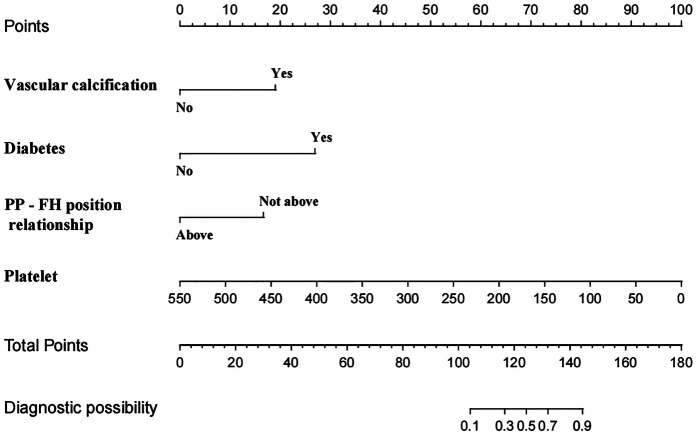
Nomogram model of femoral artery interventional access complications. PP—FH position relationship: the positional relationship between the puncture point and femoral head.

We conducted eligibility assessment for 752 patients who underwent endovascular intervention via femoral artery puncture between January 2020 and April 2025 using the hospital's electronic medical record system. After excluding 245 patients who met the exclusion criteria, 507 patients (398 males and 109 females) were ultimately enrolled in this study. Using RStudio 4.5.1, these 507 patients were randomly divided into a training set (*n* = 304) and a validation set (*n* = 203) at a 6:4 ratio. The demographic and clinical characteristics of all patients in both the training and validation sets are presented in Table 1. Among these patients, a total of 19 cases developed postoperative complications, including three cases of postoperative bleeding (0.6%), 15 cases of hematoma (3%), and one case of pseudoaneurysm (0.2%). Four potential predictors were identified based on the multivariate logistic regression analysis results (Table 2): vascular calcification [odds ratio (OR) = 7.952, 95% confidence interval (CI): 1.653–38.254], history of diabetes (OR = 18.793, 95% CI: 3.670–96.225), platelet count (OR = 0.980, 95% CI: 0.967–0.994), and positional relationship between the puncture point and femoral head (OR = 6.125, 95% CI: 1.048–35.800). The four independent predictors were then employed to develop a nomogram associated with femoral artery access complications (Figure 2).

**Table 2:** Multivariate logistic regression analysis results.

Variables	B	S.E.	Wald	P	OR	95%CI
Vascular calcification	2.073	0.801	6.692	0.010	7.952	1.653–38.254
Diabetes	2.933	0.833	12.393	0.000	18.793	3.670–96.225
PP-FH position relationship	1.812	0.901	4.048	0.044	6.125	1.048–35.800
Platelet count	-.020	0.007	8.234	0.004	0.980	0.967–0.994
Constant	−2.655	0.982	7.309	0.007	0.070	

PP—FH position relationship: the positional relationship between the puncture point and femoral head.

**Figure 3:**
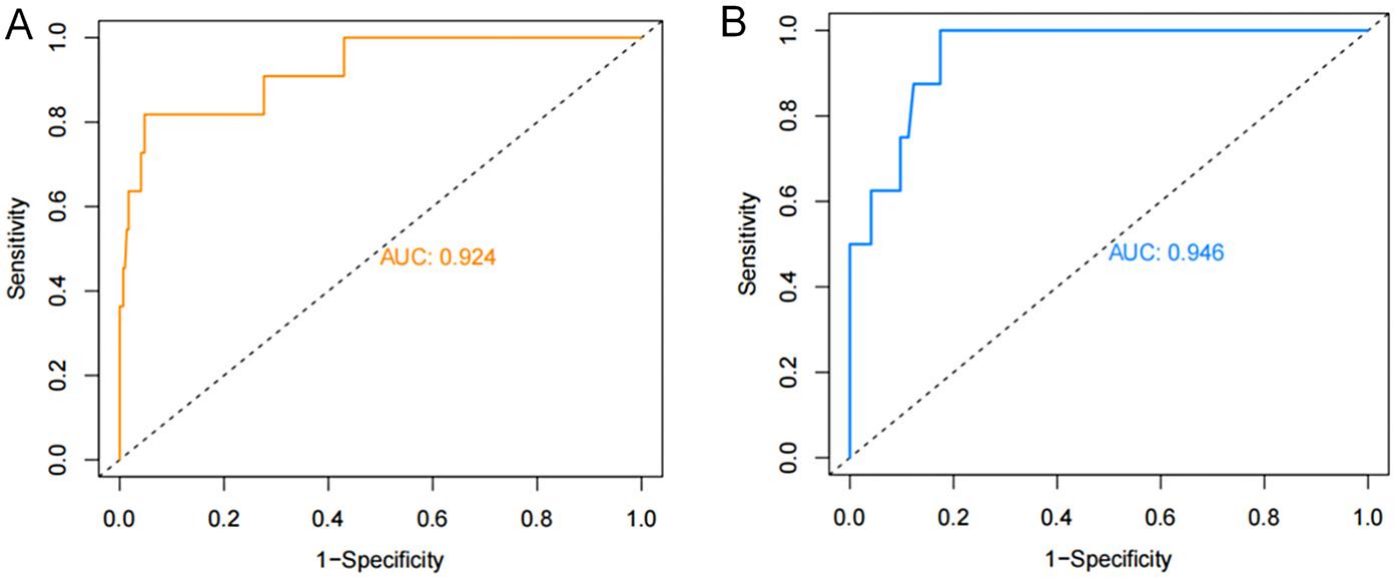
ROC validation of the nomogram prediction of femoral artery interventional access complications. The area under the curve represents the performance of the nomogram in the **(A)** training and **(B)** validation sets.

**Figure 4:**
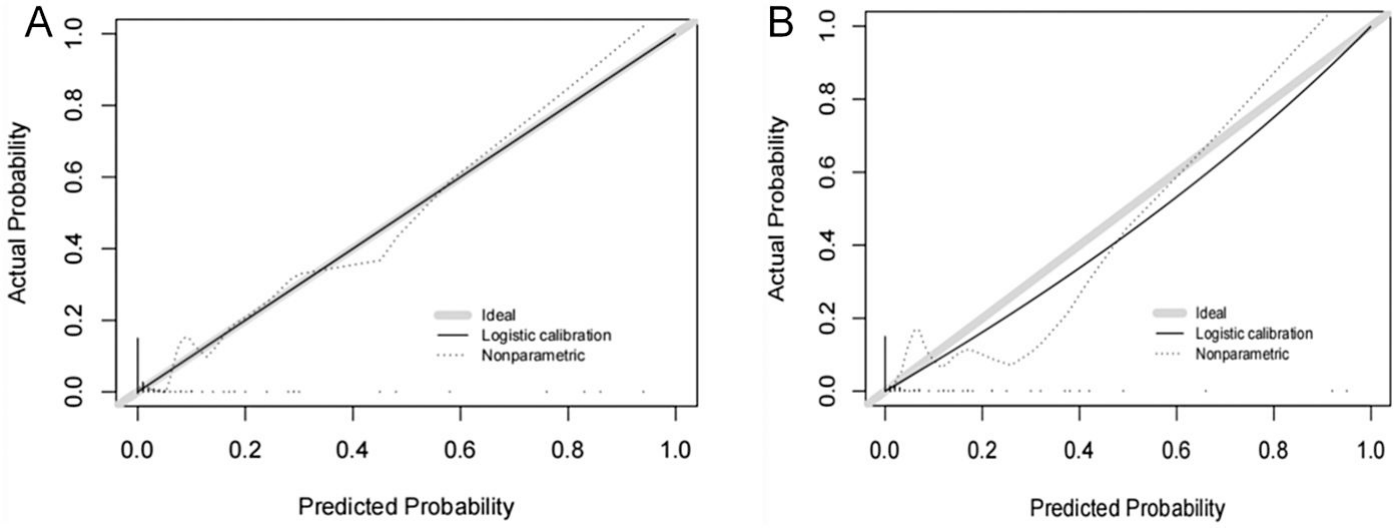
Calibration curves of femoral artery interventional access complications. The diagonal dotted line represents a perfect prediction by an ideal model. The solid line represents the performance of the **(A)** training and **(B)** validation sets, which indicated that a closer fit to the diagonal dotted line showed a better prediction.

The ROC curves were plotted and the area under the ROC curve (AUC) values were calculated in order to test the discriminatory ability of the nomogram. The AUC values for the training and validation sets were 0.924 (95% CI: 0.839–1.000) and 0.946 (95% CI: 0.896–0.997), respectively, demonstrating good diagnostic performance (Figure 3). The sensitivity, specificity, and accuracy under the optimal cutoff value were 81.80%, 95.20%, and 94.70%, respectively. Nomogram calibration was evaluated based on the calibration curves and the Hosmer-Lemeshow goodness-of-fit test (Figure 4). The calibration curves demonstrated that the nomogram's predictions of femoral artery access complications were consistent with the actual incidence. The Hosmer-Lemeshow test yielded a *p*-value of 0.8184 for the training set and 0.9983 for the validation set. Overall, the nomogram showed strong agreement between the observed outcomes and predictions.

**Figure 5:**
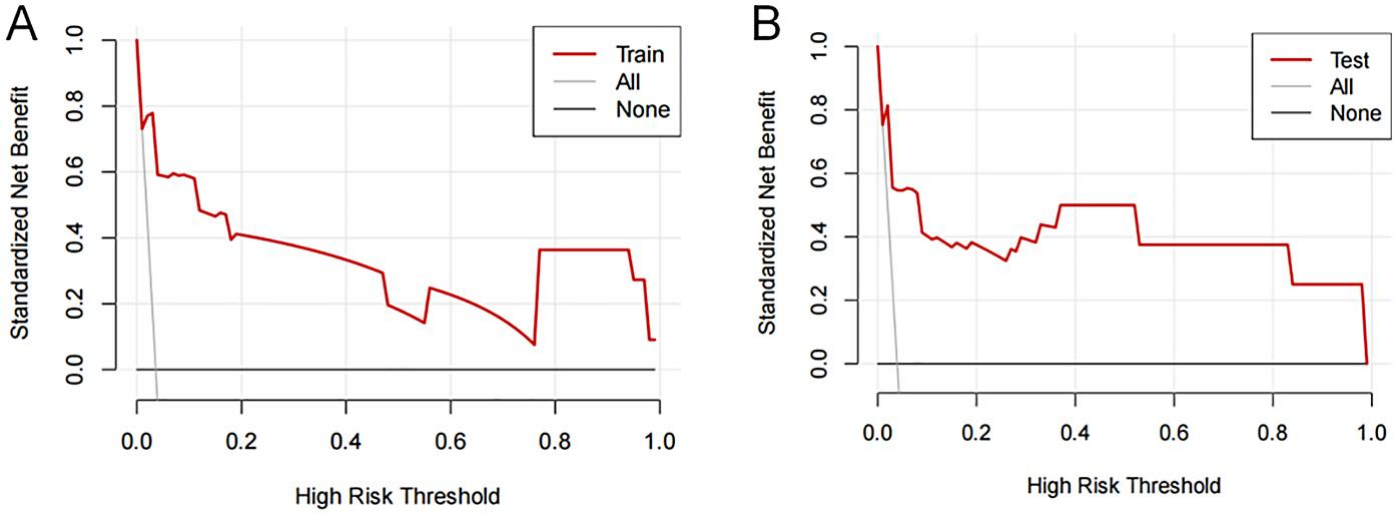
Decision curve analysis of femoral artery interventional access complications. The thick solid line represents the assumption that all patients did not develop complications after puncture. The thin solid line represents the assumption that all patients developed complications after puncture. The red line represents the risk nomogram for the **(A)** training and **(B)** validation sets.

Decision curves showed that the risk of femoral artery access complications was more accurately predicted using the nomogram when the risk threshold probability was between 3% and 100% in the present study and between 3% and 99% in the validation set (Figure 5).

## 4 Discussion

The femoral artery access complication prediction model developed in the present study is easy to implement and demonstrates strong predictive performance. It shifts the clinical approach from reactive intervention after symptom onset to proactive and targeted prevention before complications occur. ROC curves were used to evaluate the discrimination ability of the model. AUC values of 0.5–0.7 indicated low accuracy, AUC values of >0.7–0.9 represented moderate accuracy, and those ≥0.9 showed high accuracy (5).

The present study suggests that platelet count, vascular calcification, diabetes history, and relative position of the puncture point to the femoral head are independent risk factors for the complications of femoral artery access. The complication rate was 3.8% (19/507) in the 507 analyzed cases, which was lower than that reported in previous studies. The lower complication rate was primarily attributed to a systematic and precise order of operations, such as comprehensively assessing the target vessel calcification burden, thrombus distribution, and vascular tortuosity using preoperative computed tomography imaging and artificial intelligence analysis, precisely locating the spatial relationship between the puncture site and the femoral head based on intraoperative Digital Subtraction Angiography guidance to optimize the puncture path, and performing all procedures by the same experienced vascular interventional physician (6, 7). In this study, all three patients with post-procedural bleeding received continued manual compression at the femoral artery puncture site for 30 min, followed by the application of an elastic compression bandage for 6 h. Patients who developed a puncture site hematoma did not receive any specific additional intervention. For the patient diagnosed with a pseudoaneurysm, follow-up color Doppler ultrasound performed 24 h postoperatively revealed a small aneurysm sac diameter, and thus no specific treatment was administered.

Research indicates that the optimal femoral artery puncture site is located at the midline level of the femoral head, which serves as a key bony landmark to indicate femoral artery course. The central plane of the femoral head closely corresponds to the anatomical course of the femoral artery. Schnyder et al. (8, 9) found that the femoral artery bifurcation is located at or slightly below the level of the femoral head midline in 98% of patients. Puncture at the level of the femoral head midline can avoid retroperitoneal bleeding caused by high-level puncture into the external iliac artery (10). Ahn et al. found that the safe distance between the puncture point and the femoral artery bifurcation is the largest and the risk of complications is the lowest when the puncture point is at the level of the femoral head midline. Moreover, femoral artery calcification is typically concentrated at the arterial bifurcation, whereas the vascular wall at the level of the femoral head midline generally exhibits minimal calcification (10). Puncturing calcified vessels often results in needle tip deviation or incomplete vessel entry, increasing the risk of repeated puncture attempts and subsequent hematoma formation (11). However, it is difficult to monitor the long blood vessels on the same interface due to the probe scanning range limitations. The femoral head provides a firm bony support for post-procedural compression at the vascular puncture site, facilitating effective vessel closure through compression devices or manual pressure, thereby significantly reducing time to hemostasis and minimizing vascular displacement (7, 12–14). In addition, the distance between the femoral nerve and the blood vessels at this level is the largest (1–2 cm lateral), which can prevent nerve damage (15).

Long-term hyperglycemia exacerbates vascular lesions through multiple pathological mechanisms, including inducing endothelial cell dysfunction, increasing vascular permeability, and promoting low-density lipoprotein cholesterol (LDL-C) to penetrate the intima, oxidize, and form early atherosclerotic lesions (16). The hyperglycemic environment produces excessive reactive oxygen species, accelerating LDL-C oxidation and further promoting atherosclerosis progression (17). In addition, persistent hyperglycemia enhances the accumulation of advanced glycation end products (AGEs) by activating the Receptor for Advanced Glycation End Products (RAGE) receptor pathway. This condition increases collagen cross-linking and reduces elastic fiber content in the vascular wall, promoting vascular sclerosis (18–20). It simultaneously stimulates the proliferation and migration of vascular smooth muscle cells, thereby accelerating vascular calcification (20). The formation of calcified plaques significantly increases vascular fragility and the risk of vascular tearing or rupture during puncture operations (21, 22), weakens the vascular contraction and hemostasis ability, and reduces the efficiency of vascular closure devices (VCDs).

AGEs also impair the function of endothelial progenitor cells, delaying vascular repair and resulting in prolonged hemostasis and extended compression time. This, in turn, increases the risk of a hematoma and pseudoaneurysm formation at the puncture site. Additionally, hyperglycemia can directly activate platelets, stimulate thromboxane A2 release, and enhance platelet aggregation and adhesion (23–25). Studies have shown that platelet reactivity in diabetic patients is significantly higher than that in non-diabetic patients (26). The accompanying endothelial damage reduces the secretion of nitric oxide and prostacyclin and induces abnormalities in the morphology and function of red blood cells and platelets, leading to a hypercoagulable state of blood (27–29). In this context, local hemodynamic changes after femoral artery puncture can easily induce thrombosis at the puncture site, thereby causing lower limb arterial embolism and circulatory disorders. It is worth noting that the peripheral nerve and microvascular lesions associated with diabetes can cause local tissue perfusion insufficiency and nutrient deficiency, significantly delaying the wound healing process and increasing the risk of complications, such as wound dehiscence and infection.

Vascular calcification can be classified into atherosclerosis-related intimal calcification and non-occlusive medial calcification based on its anatomical location, the latter primarily contributing to increased vascular stiffness and reduced compliance. Clinical studies have confirmed that severe vascular calcification can significantly impact VCD effectiveness and is an independent risk factor limiting successful VCD application (30). Additionally, calcified plaques create irregularities in the vessel wall, increasing the risk of intimal tearing and plaque rupture, which may lead to thrombosis or lipid embolism. The associated luminal stenosis further contributes to thrombus formation by disrupting normal hemodynamics (31, 32). Studies have shown that ultrasound guidance can increase the success rate of vascular closure device (VCD) deployment and reduce the incidence of complications (33). Calcified plaques can interfere with the accurate positioning of blood vessels using ultrasound (34) and cause the course of blood vessels to be relatively fixed, increasing the difficulty of the operation. Severe calcification in specific areas, such as the aorta and dialysis access vessels, is more likely to cause ischemia or even fatal complications. These risks are further heightened in the presence of hypertension or coagulation disorders (35, 36). Therefore, calcification of the anterior femoral artery wall has been reported as an independent risk factor for adverse events, such as vascular puncture complications and vascular suture device failure (37).

Therefore, appropriate perioperative preventive measures can be implemented, including establishing and strictly adhering to a standardized operating procedure for femoral artery puncture, improving preoperative evaluations, planning surgical and puncture procedures based on patient CT and three-dimensional reconstruction images, maintaining adequate glycemic control, enhancing theoretical knowledge and technical skills training, and standardizing postoperative monitoring, so as to minimize surgical trauma and reduce the incidence of severe postoperative complications.

The present study had several limitations. As a single-center retrospective analysis investigation, it was subject to inherent biases. Additionally, the low incidence of complications and limited sample size underscore the need for further studies with larger cohorts. The absence of long-term follow-up data and the omission of certain potential risk factors may render the conclusions somewhat limited and subjective. The sample size of the constructed model was relatively small, and only internal validation methods were used for verification. Therefore, future large-sample, multicenter prospective studies are needed to further revise and optimize the model to improve its generalization ability and better meet the clinical needs.

## 5 Conclusions

Platelet count, vascular calcification, positional relationship between the puncture site and the femoral head, and diabetes are the independent risk factors for femoral artery access complications. The nomogram prediction model developed based on these risk factors demonstrated a strong predictive value.

## Data Availability

The datasets presented in this study can be found in online repositories. The names of the repository/repositories and accession number(s) can be found below: National Whole Population Health Protection Information Platform—Medical Research Registration and Record Filing Information System.
